# Global climate governance remains resilient under Trump 2.0

**DOI:** 10.1007/s43508-025-00131-x

**Published:** 2025-11-17

**Authors:** Yixian Sun, Yitong Ye

**Affiliations:** Department of Social and Policy Sciences, University of Bath, Bath, UK

**Keywords:** Climate action, Global governance, Net zero, Polycentricity, The US, Trump

## Abstract

The US is retreating from climate action under the second Trump administration, as demonstrated by the country’s withdrawal from the Paris Agreement, cancellation of its international climate finance program, and also various policies supporting oil and gas exploration. How is global climate governance impacted by Trump’s anti-climate policy, and how do different actors react to this shift? By assessing the changing dynamics in multilateral and transnational arenas of climate governance, we argue that Trump’s anti-climate policy cannot reverse net zero transitions across the globe due to polycentric climate governance. At the multilateral level, the Paris Agreement remains resilient to the US withdrawal, demonstrated by the continuous support of other major emitters. At the transnational level, subnational entities and businesses have strong incentives to continue net zero transitions to respond growing impact of the climate crisis. In the Global South, many countries have accelerated net zero transitions due to accessibility to cheaper technologies and financing from new sources especially China. Our analysis suggests that Trumpism has limited impact on the polycentric system of global climate governance where various actors with strong incentives to take climate action operate through institutions and networks across multiple scales.

## Introduction

Our global governance system has undergone a fundamental shift away from the liberal international order formed at the end of the Cold War (Lake et al., 2021). While the growing influence of emerging economies and the rise of populism in Western democracies are deemed key drivers, the second presidency of Donald Trump has undoubtedly accelerated this shift by introducing a range of radical polices. Climate change is arguably one of the most important examples. Since the beginning of 2025, global climate governance has been under severe threat from the US’ retreat from climate action, including its withdrawal from the Paris Agreement and support for fossil fuels.^1^ Trump’s “America First” policy will significantly delay net zero transitions in the world’s largest economy, and also undermine global efforts to combat climate change. Given the nature of climate change as a super wicked problem (Levin et al., 2012), Trump’s anti-climate policies and discourse have raised serious concerns over the prospects of keeping the global temperature rise to 1.5 °C and avoiding climate catastrophes.

How does Trumpism reconfigure global climate governance? In this article, we assess the impact of Trump’s policies on global climate governance in both short and long terms. Our analysis focuses on policy responses of different actors and endurance of international institutions. We argue that, despite some immediate shocks caused by the US’ retreat from climate action, the existing global climate governance system characterized by polycentricity remains resilient. In other words, Trumpism can hardly reverse ongoing net zero transitions across the globe, because states and non-state actors are increasingly aware of growing impact of climate change and increasing benefits of taking climate action. In the rest of the article, we first analyse Trump’s anti-climate policies and their immediate impact. We then introduce our theoretical argument and use evidence from multiple data sources to illustrate the continuity of climate action around the world.

## Trump 2.0’s climate rollback and its consequences

The return of Donald Trump to the White House in 2025 marks a significant rollback of climate action in the US. While Trump’s first administration (2017–2021) already attempted to reverse course on the US’ climate action, many measures were regulatory rather than structural, and thus largely manageable and reversible by subsequent administrations (Jotzo et al., 2018). However, since returning to office in 2025, Trump’s “America First” policy started a new round of climate rollback with far more speed, coherence, and determination than during his first term.

On the first day of his second presidency, Trump enacted a series of executive orders to withdraw the US from the Paris Agreement and also suspend climate-related provisions of the Inflation Reduction Act (IRA). He also directed the Environmental Protection Agency to reconsider the scientific and legal determination that provides the basis for regulating greenhouse gas emissions under the Clean Air Act (EPA, 2025). Moreover, references to “climate change” and “environmental justice,” have been removed from federal websites, and all programs that support climate adaptation, assessments, and risk disclosure have been defunded or halted (BBC, 2025). These moves signal a systematic shift towards structural dismantling, targeting not only climate regulations but the underlying legal, scientific, and institutional foundations on which climate action depends. Below we highlight three areas of Trump’s anti-climate policy and consider the harm they can cause to global climate action.

### Withdrawal from multilateralism

First, as the US is out of the Paris Agreement, the multilateral system governing climate change faces a serious challenge. A key feature of climate change as a wicked problem is the lack of central authority, so countries must make arrangements to ensure cooperation and avoid free-riding (Levin et al., 2012; Puppim de Oliveira & Qian, 2023). The Paris Agreement was designed as an adaptive and legally-binding framework to address this collective action problem (Falkner, 2016). It requires all members to submit their Nationally Determined Contributions (NDCs) every five years, thereby embedding national pledges in an international mechanism of accountability. By allowing member states to decide their own targets, the Agreement was able to win the support of all major emitters, especially the US which was strongly against any targets imposed internationally like the Kyoto Protocol. Additionally, it establishes a ratcheting-up mechanism requiring countries to progressively increase targets in their new NDCs. This design can encourage countries to increase their ambition over time, with the expectation that all other parties will also enhance their engagement, thereby fostering collective action (Falkner, 2016).

Rather than simply a symbolic rejection of multilateral cooperation, the formal withdrawal of the US in 2025 creates a fundamental challenge to the credibility and stability of the existing multilateral processes. As the Paris Agreement is based on member states’ own commitments, the exit of the world’s largest economy and second largest emitter sent a strong signal to the world that participation in this framework can be easily reversed. This, in turn, could trigger a domino effect, prompting countries sceptical of climate change to also withdraw or to adopt far less ambitious climate targets (Diringer, 2017). In other words, Trump’s climate denialism risks leading other states to opt for incremental or symbolic pledges even if they stay in the Paris Agreement. This may significantly reduce the likelihood of achieving the global climate goals while undermining international justice.

As such, the confidence of other countries in multilateral climate cooperation is likely to weaken, and more burdens need to be taken by countries with less historical responsibility for climate change. Hence, while the US’ attempt to withdraw from Trump’s first presidency had shown the system’s resilience, the actual withdrawal in 2025 poses a serious threat to the future of multilateral climate governance system and the spirit of collective action that this system aims to produce.

### Reversal of green industrial policy

A second major area of Trump’s anti-climate policy is the reversal of the US’ green industrial policy, particularly the dismantling of the IRA enacted by the Biden Administration, and the reorientation of federal support toward fossil fuels. The IRA is the largest public investment programme in US history to promote sustainability transition by mobilizing approximately $370 billion for the development and adoption of renewable and zero-emission technologies. The IRA stands as a milestone, as it delivers, for the first time, a legislated and comprehensive federal framework for climate action (Bang, 2024). However, once starting his second presidency in January 2025, Trump issued an executive order to suspend the disbursement of tax credits and funds authorized under the IRA. While a complete repeal of the IRA remains in question, the Trump administration is increasingly hollowing out the Act’s fiscal capacity to delay future climate-related appropriations.

This radical policy reversal has been reinforced by the administration’s reasserted commitment to expanding domestic oil, gas, and coal production, framed as an agenda for “energy dominance” and “economic security” (White House, 2025). The removal of clean energy investments, combined with financial support for fossil fuels, would significantly reduce economic benefits of decarbonization in the US, and thereby deepen the structural lock-in of a high-carbon economy.

The impact of Trumpism goes beyond even federal policy. Following Trump’s denial of climate change, Republican-run states start to ramp up legal and political challenges to prevent businesses from pursuing their net zero transitions. This is particularly exemplified by a letter issued in August 2025 by 23 Republican state attorneys general to the Science Based Targets initiative (SBTi) – a global standard-setter on corporate climate action, demanding disclosures about its governance and membership on the grounds that companies’ voluntary adoption of SBTi’s net zero targets might contravene antitrust, consumer protection, or other statutory obligations (Grogan-Fenn, 2025; Iowa Attorney General, 2025). Such threats from subnational governments to non-state climate action are unprecedented and may have large consequences by deterring US-based companies from supporting climate action.

### Suspension of support for developing countries

Another key area of Trump’s policy is his suspension of assistance for developing countries’ climate action. Upon taking office, Trump imposed a 90-day freeze on foreign development assistance and announced the closure of U.S. Agency for International Development (USAID). This led to the termination of over 150 contracts and grants (roughly $1.2 billion) related to climate change (Schonhardt, 2025). The Trump administration also rescinded the $4 billion previously committed to the Green Climate Fund and announced its withdrawal from the Climate Loss and Damage Fund (POLITICO, 2025). As the US was the largest supplier of international climate finance, the end of USAID will no doubt interrupt green transitions in many developing countries.

Behind this strategy is a shifting philosophy of US foreign policy, moving away from cooperative leadership based on the provision of global public goods towards coercive influence exercised through punitive measures, especially tariffs. Assistance to developing countries, including climate finance, has long served both as a moral obligation and a strategic instrument of US diplomacy, seeking to stabilize regions, build partnerships, and embed national norms in global governance (Hicks et al., 2008). In contrast, Trumpism embodies a turn to the “America First” economic nationalism (Ettinger & Collins, 2025). According to Trump, international commitments, whether climate aid, multilateral institutions, or trade rules, are only constraints on US sovereignty and can only allow other countries to “exploit” US resources. In other words, climate finance is a zero-sum transaction that only benefits other countries at America’s expense. Hence, Trump has introduced a major shift in US foreign policy, which relies on bullying and is dismantling the post-Cold War international system.

Financial support from developed countries is a critical channel through which low- and middle-income countries access technologies, build institutional capacity, and manage the escalating costs of climate-induced loss and damage. As the US has been the most important contributor of international climate finance, developing countries’ capacity to combat climate change would be significantly weakened. More fundamentally, this change would deepen global structural inequality, marginalizing countries and communities most vulnerable to climate risks while absolving the world’s largest historical emitters of its responsibility.

Moreover, Trump declared a national emergency over the U.S. trade deficit and announced plans to impose a universal baseline tariff of at least 10% on all imports. The resulting trade tensions intensified into a retaliatory spiral, especially between the US and China, the principal supplier of clean technologies. This sharp escalation has raised the costs of clean energy components in the US ranging from solar panels and batteries to electric vehicles.

In summary, Trump’s second presidency has undoubtedly brought significant challenges to climate action both in the US and worldwide. Despite the damage made and worrying trends caused by Trumpism, the extent to which the US retreat can impact global climate governance requires a careful assessment. In the next section, we argue that the US’ anti-climate policy can hardly destroy today’s climate governance system due to the system’s polycentric feature as shown by the existence of many different actors, institutions, and incentives.

## Resilience of global climate governance due to polycentricity

In light of slow progress and deadlocks in intergovernmental processes, global climate governance over the last two decades has experienced a polycentric turn, characterised by growing participation of subnational governments, businesses and civil society groups and transnational networks of these actors (Bulkeley et al., 2014; Jordan et al., 2018). The concept of polycentricity effectively captures the emergence of multiple and overlapping centres of authority that operate across different governance scales (Ostrom, 2010). From this perspective, climate governance is not confined to the Paris Agreement or the UN Framework Convention on Climate Change (UNFCCC, 2025), but carried out by a diverse array of actors operating across various levels who formulate and implement rules, norms, and innovations (Andonova et al., 2009; Hoffmann, 2011; Jordan et al., 2018). Empirical evidence suggests that transnational governance initiatives led by non-state and subnational actors have complemented, and at times even substituted for, states’ efforts in advancing climate action (Bäckstrand et al., 2017; Andonova & Sun, 2019; Chen & Xie, 2023). Hence, with polycentricity becoming a defining feature of global climate governance, many different actors and institutions can become agents of change to exert influence across various scales.

This polycentric structure endows global climate governance with a high degree of resilience, with a strong capacity to adapt to external shocks. This is because when authority is distributed across multiple centres in a complex system composed of diverse networks and clusters, the system no longer depends on any single actor, or process, nor can a small number of actors exert dominant influence over the entire regime (Morin & Kim, 2025). This means the diffusion of authority in a polycentric system constitutes a safety net to mitigate the risk when one actor fails to address a policy problem. In other words, the system is poised to minimize the impact caused by shifting policy of individual countries even if such a shift is from a major player. In the case of a US retreat, other actors within the polycentric climate governance system, including other major states, subnational networks, business coalitions, can step up to sustain net zero transitions.

Additionally, as the impact of climate change continues to intensify, actors are increasingly aware of costs and benefits of climate action for them. In this context, climate politics is less centred around solving a collective action problem, but more on addressing distributional effects of climate change (Aklin & Mildenberger, 2020; Colgan et al., 2021). Accordingly, actors benefiting from climate action are likely to strengthen their action over time, while others seeking such benefits or wanting to avoid costs of inaction would have incentives to support transition. Today climate change is no longer simply an environmental policy issue but deeply embedded into many countries’ geoeconomic strategies (Meckling, 2025). For this reason, the impact of Trump’s policy on other actors’ interest in taking climate action may be very limited.

Taking into account today’s polycentric system of global climate governance, it is reasonable to expect that global net zero transitions cannot be easily reversed by Trump’s anti-climate policy. Instead, various institutions and initiatives supporting climate action should continue to operate across multiple scales and entrenched incentives of many actors in supporting climate action are likely to exist.

## Trumpism’s limited impact on global climate governance

To illustrate our argument, we turn to recent evidence of global reactions to Trump’s anti-climate policy, which shows a resilient system of climate governance. As the impact on behavioural change measured by emissions or funding allocation can be only observed in the long term and are likely mediated by other factors, our analysis focuses on actors’ policy responses and institutional endurance. That said, the evidence shown below suggests the continuation of global net zero transitions.

### Continuous international cooperation

At the multilateral level, climate action and cooperation persist despite a headwind caused by Trump’s policy. The Paris Agreement and the UNFCCC continue to retain support from the rest of the world. Until now, no other member state has expressed interest in following the US to withdraw from the Paris Agreement. On the contrary, most countries continue their support for the Agreement by updating their NDCs. In July 2025, two major emitters – China and the EU – committed to submit their enhanced 2035 NDCs (NDCs 3.0) before COP30 and to demonstrate leadership together in advancing global net zero transitions in the context of the US retreat (European Council, 2025).

More recently, on 24 September 2025, Chinese President Xi Jinping announced the country’s updated NDC targets, which include achieving 7–10% of emission reductions below peak levels by 2035, increasing the share of non-fossil fuels in total energy consumption to over 30%, and expanding the installed capacity of wind and solar power to over six times the 2020 levels (Xinhua, 2025). Although the ambition level of these targets remains debatable, it is the first time China has promised an absolute limit on its greenhouse gas emissions and the country is likely to overperform if it can continue its current speed of clean energy transition (Patel & Evans, 2025). Several other major emitters have also expressed their continuous support for the Paris Agreement despite the US withdrawal. For instance, Brazil has pledged 59–67% emissions reduction below 2005 levels by 2035 in its updated NDCs and has shown its intention to use its COP 30 presidency to keep global ambition aligned with the Paris goal. Table 1 summarizes the targets announced by major emitters for their NDCs due in 2025.

**Table 1:** Major emitters’ updated targets in NDC 3.0 (as of November 5, 2025)

Country/region	Updated mitigation targets by 2035	NDC 3.0 status
China	7–10% reduction below peak levels by 2035	Submitted
EU	An indicative target range of 66.25%-72.5% emissions reduction by 2035 below 1990 levels was proposed, but has not yet been adopted internally	Submitted
United Kingdom	81% below 1990 levels by 2035, net-zero by 2050	Submitted
Japan	60% reduction below 2013 levels by 2035	Submitted
Australia	62–70% reduction below 2005 levels by 2035	Submitted
Russia	65–67% reduction below 1990 levels by 2035	Submitted
Brazil	59–67% reduction below 2005 levels by 2035	Submitted
Canada	45–50% reduction below 2005 levels by 2035	Submitted
South Africa	Keeping annual GHG emissions in the range of 320–380 Mt CO₂-equivalent in 2031–2035	Submitted
India	Not yet announced	Not yet submitted, but likely to submit around COP30

(Data source: climate action tracker (2025)

**Fig. 1:**
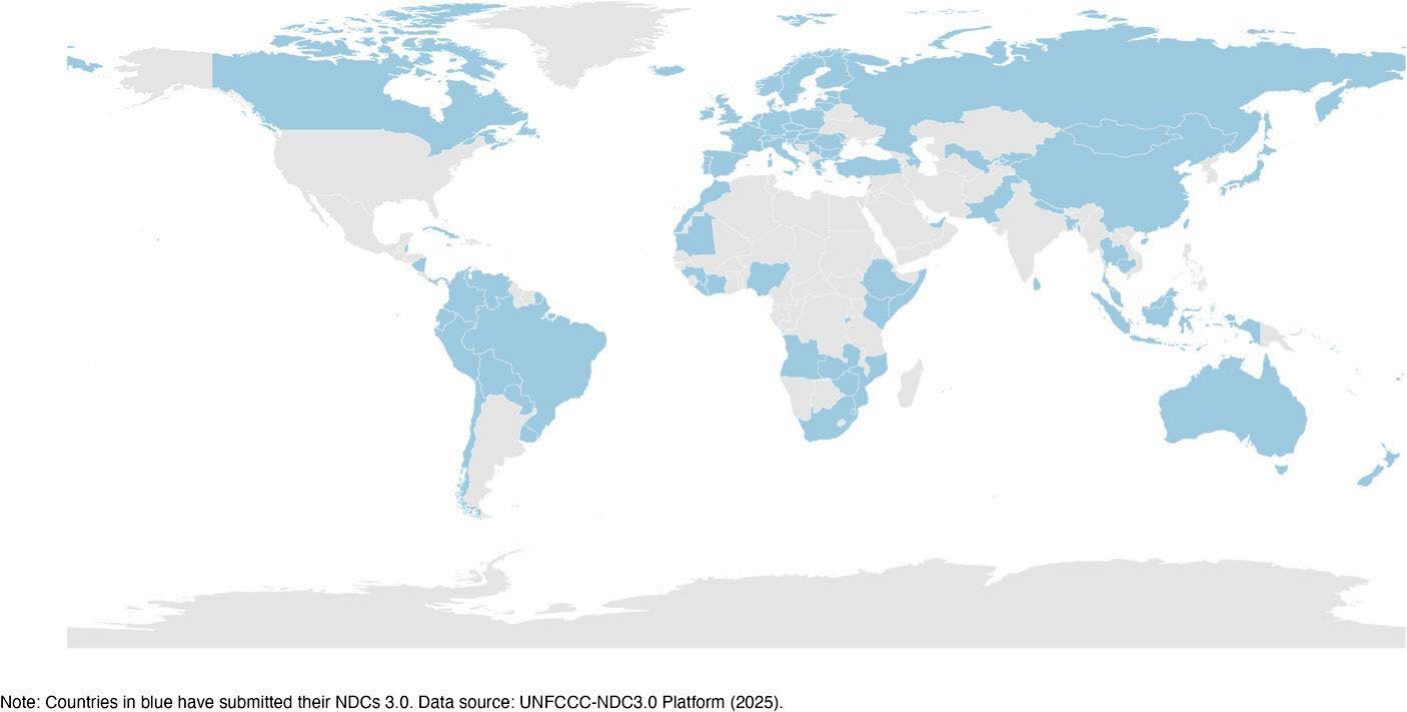
Countries having submitted NDCs 3.0 before COP30

As of 9 November 2025, 108 parties (excluding the US) have officially submitted their 2035 NDC targets (See Fig. 1).^2^ Together, these countries inclduing major emitters such as China and the EU, account for 65.3% of global emissions and 55.3% of the global population. Other major emitters, notably India, is expected to submit their NDCs around COP 30. The continuous engagement of the international community in upgrading its climate targets shows the resilience of the Paris Agreement and multilateral climate cooperation more broadly. Although the US’ withdrawal may undermine some states’ ambition in their climate action, most states are still determined to strengthen their climate action as required by the Paris Agreement’s ratcheting up mechanism. As states become increasingly aware of the impact of climate change and urgency of decarbonization, the multilateral climate governance system is unlikely to be derailed by Trumpism.

### Subnational and non-state climate action in the US

**Fig. 2:**
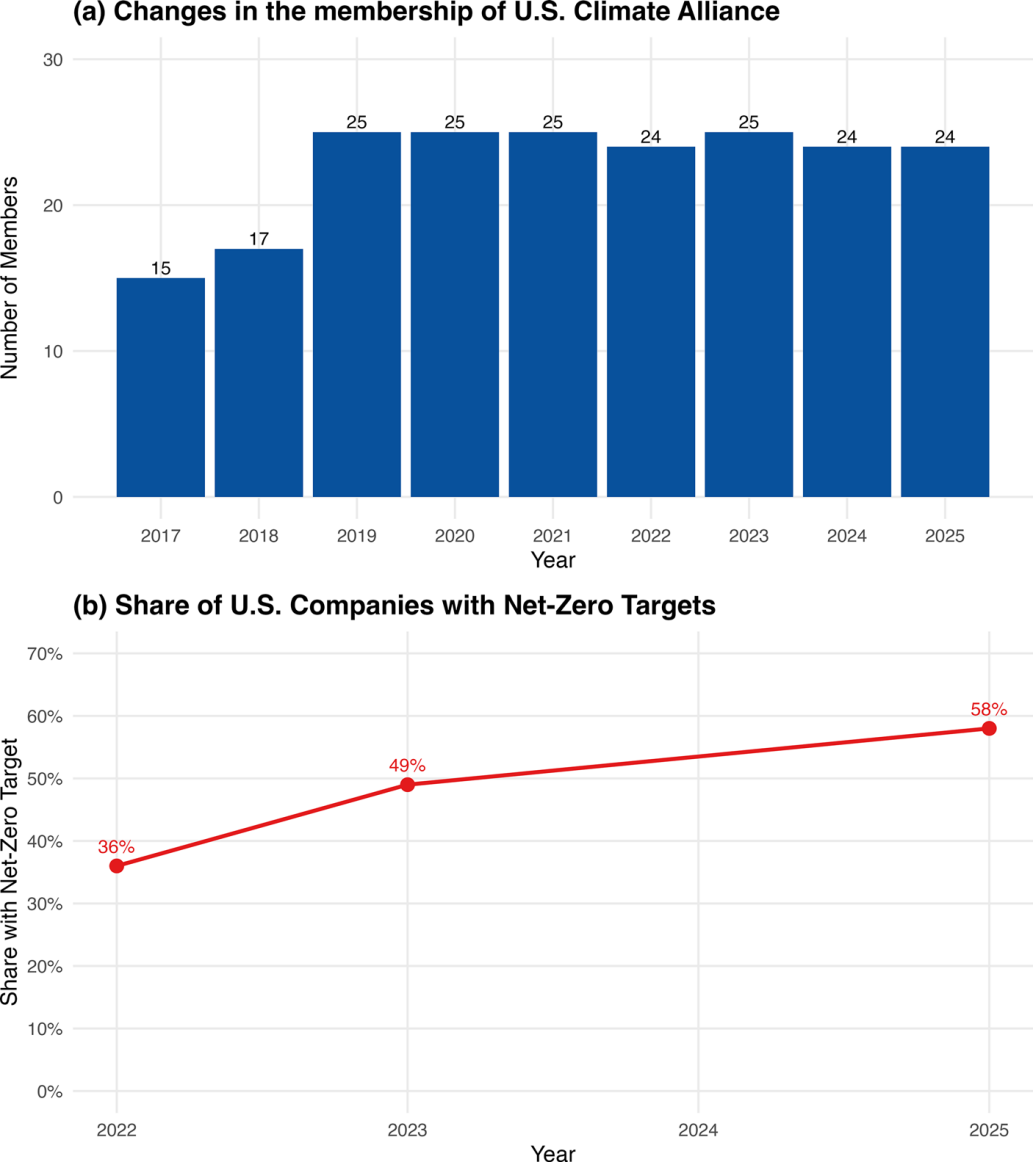
Dynamics of U.S. state and corporate climate action 2017–2025. (Note: Panel (**a**) is compiled from the annual reports of the U.S. Climate Alliance; Panel (**b**) is based on Net Zero Tracker Stocktake in 2022-2025, covering the 2,000 largest publicly traded companies worldwide. Data for 2024 are unavailable due to missing data)

While the policy chosen by the second Trump administration has undermined climate leadership of the US national government, initiatives led by subnational entities and businesses continue to advance green transition in the country. In fact, subnational and non-state actors in the US have always been key proponents of transnational climate governance, driving climate action in the country and worldwide. At the state level, 24 states and territories have formed the US Climate Alliance, representing nearly 60% of the national economy. This alliance has collectively reaffirmed its commitment to a net-zero target, planning to reduce emissions by 50–52% below 2005 levels by 2030, regardless of the policy changes at the federal level (U.S. Climate Alliance, 2025). Figure 2a shows the evolution of the alliance membership, which remained stable despite Trump’s climate rollback.

For many years, state governments have demonstrated strong leadership in addressing climate change. Some states, such as California, Washington, and Massachusetts, have initiated renewable portfolio standards, implemented cap-and-trade systems, and expanded electric vehicle mandates (U.S. Climate Alliance, 2024). Interestingly, even some Republican-leaning states have continued to drive clean energy expansion for economic and market reasons. Texas, the largest oil- and gas-producing state, has simultaneously become a national leader in renewable generation, accounting for over 26% of total U.S. wind capacity in 2022 and attracting more than $21 billion in solar investment as of early 2023 (Texas Comptroller of Public Accounts, 2023).

At the municipal level, city networks also play a key role in sustaining climate action in the US. A prominent example is the C40 Cities Climate Leadership Group, which includes 14 major U.S. cities. In response to Trump’s anti-climate policy, mayors in the network indicated that cities would remain bastions of climate progress, committing to continued emissions reduction and the pursuit of a green economy (C40 Cities, 2025).

Additionally, many businesses in the US have taken the lead in climate action to pursue benefits from net zero transition or reduce risks from climate change. Over the last decade, an increasing number of US-based companies have adopted net-zero targets, even during the early phase of Trump’s second term. Figure 2b shows an upward trend in the proportion of US-based companies with net zero pledge among 2000 largest companies worldwide. Therefore, bottom-up initiatives from subnational and non-state entities have been a key driving force in keeping the US’ climate action on track under Trump 2.0.

### New incentives and financing for global net zero transitions

While the US has abandoned its international climate finance programme, developing countries now possess new and stronger incentives to embrace net-zero transitions due to access to cheaper green technologies. The recent boom led by consumer choices across several Global South countries is a telling example. In Nigeria, the savings from reduced diesel imports would allow solar panels to pay back their cost within six months of operation; in Sierra Leone, the imported solar panels could generate electricity equivalent to 61% of the country’s 2023 output if fully installed; and Pakistan has recorded one of the fastest expansions in solar deployment in history by importing 16 Gigawatts of solar panels during 2024 (Jones, 2025). This rapid transition suggests that clean energy is now accessible in the Global South where consumers and businesses can have strong economic incentives to support low-carbon development. In fact, for many low- and middle-income countries, the value of clean energy lies not only in alleviating energy shortages but also in promoting socioeconomic development through more reliable, cleaner, and affordable electricity access.

Moreover, the US failed to lead the developed country group to deliver the promise made by Obama to mobilize USD 100 billion climate finance per year by 2020 (Qian et al., 2023). This unfulfilled commitment has undermined the US’ climate leadership in eyes of developing countries. Meanwhile, new sources of financing are emerging – mostly driven by China – to support net zero transitions in the Global South. As a developing country, China has voluntarily provided during 2015–2021 an estimated USD 3 billion annually to support climate action in developing countries, placing China among the world’s fifth largest climate-finance provider (Liu et al., 2025). In addition to being a world leader in green technologies, China has also pledged massive overseas investments in green technologies – up to USD 71.6 billion – especially since 2022 (Xue & Larson, 2025). China has made this strategic move towards financing green development worldwide in order to leverage its comparative advantages in clean technologies and maximize economic benefits. At the same time, it can create win-win opportunities for many developing countries through job creation and technology transfer.

**Fig. 3:**
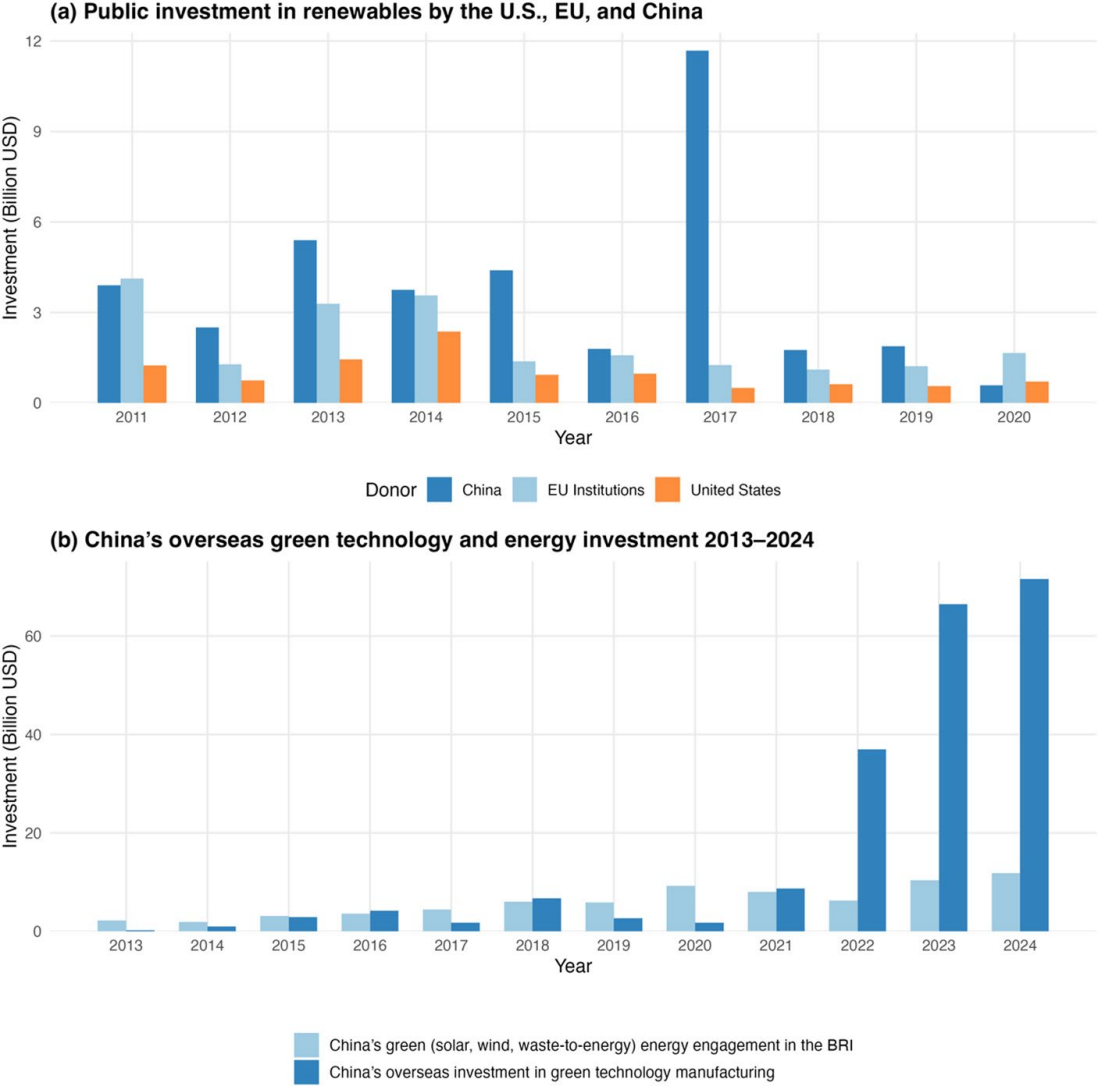
Global and Chinese trends in green investments. (Data sources: IRENA, 2025; Xue and Larson, 2025; Nedopil, 2025.)

The recent momentum in energy transition in the Global South and China’s overseas green investments suggest a major shift in the global landscape of financing for net zero transitions, which suggests the US is no longer a solution provider. As shown in Fig. 3a, this shift has started already a decade and half ago as the US’ overseas investments in renewables have been outpaced by China and the EU. More importantly, since 2022, China has significantly increased its investments around the world, especially through the Belt and Road Initiative (BRI), in clean energy production and green technology manufacturing (see Fig. 3b). Hence, Trump’s decision to stop the US’ international climate finance can only have a limited impact on global net zero transitions, since growing demand for green transitions in the Global South will still be supported by technologies and capital from China. Instead, Trump’s denial of climate change may only further strengthen China’s dominance in global cleantech supply chains.

## Conclusion

Under Trump’s second presidency, the US has adopted a series of policies to delay net zero transitions both domestically and internationally. While Trump’s radical anti-climate approach has negative consequences on climate action, we argue that the existing system governing climate change characterized by polycentricity remains resilient to Trumpism. This is because the system consists of a range of state and non-state actors operating through various institutions and networks across multiple scales and many actors’ incentives to take climate action continue to increase due to growing impact of climate change. To illustrate our argument, we analysed three key areas of Trump’s anti-climate policy: withdrawal from the Paris Agreement, reversal of green industrial policy, and suspension of international climate finance. While Trump’s policy has undoubtedly had a negative impact on global climate governance, recent evidence suggests that such impact is limited. Despite the US retreat, many other actors – including major emitters and developing countries as well as subnational and non-state actors in the US – still have a strong interest in strengthening their climate action. While long-term implications of Trump’s anti-climate policy can only be seen in the future, our analysis shows promise of the polycentric system of global climate governance, which is likely to sustain net zero transitions around the world.

## Footnotes

1 We use a broad definition of ‘global climate governance’ by including formal and informal rules, institutions and decision-making processes across different scales to address climate change.

2 Altough the US under the Biden Administration submmited their NDCs 3.0 in December 2024, we do not count it here due to Trump's withdrawal from the Paris Agreement in early 2025.
